# Synthesis and characterization of Co, Ni, Zr and Cu MOFs based on 1,4-naphthalenedicarboxylic acid linker for hydrogen generation

**DOI:** 10.1038/s41598-025-27518-4

**Published:** 2025-11-25

**Authors:** Mostafa Farrag

**Affiliations:** Nanoclusters and Photocatalysis Laboratory, Chemistry Department, Faculty of Science, Assiut University, Assiut, 71516 Egypt

**Keywords:** Non-noble metal clusters, 1,4-Naphthalenedicarboxylic acid, Hydrogen generation, Sodium borohydride hydrolysis, Base additive effect, Chemistry, Materials science

## Abstract

Cobalt-, nickel-, copper-, and zirconium-cluster-based metal–organic frameworks (MOFs) were synthesized via the solvothermal method using 1,4-naphthalenedicarboxylic acid (1,4-NDC) as the organic linker. The synthesized MOFs were characterized by PXRD, XPS, TEM, and the BET model. Powder X-ray diffraction (PXRD) confirmed that the MOFs possessed highly crystalline structures. These MOFs were utilized as innovative catalysts to produce hydrogen through the hydrolysis of sodium borohydride (NaBH₄), a promising hydrogen source for energy applications. Among them, Co(1,4-NDC) and Ni(1,4-NDC) catalysts exhibited the highest catalytic activity, producing the maximum hydrogen volume within 5.1 and 6.0 min of stirring at room temperature, respectively. The high catalytic activity is attributed to the small particle sizes of the cobalt and nickel clusters in the MOFs, as confirmed by TEM analysis, and to their large surface areas, with Co(1,4-NDC) and Ni(1,4-NDC) exhibiting specific surface areas of 820 and 770 m²/g, respectively. The effects of catalyst weight and reaction temperature (30–60 °C) were investigated. Co(1,4-NDC) and Ni(1,4-NDC) showed high hydrogen generation rates (HGR) of 1777 and 1333 mL H₂ g⁻¹ min⁻¹ at 60 °C, respectively. The apparent activation energy of NaBH₄ hydrolysis over Ni(1,4-NDC) was 33.94 kJ/mol, which is lower than that of many MOFs with different linkers reported in the literature. The presence of a base (NaOH) increased the hydrogen generation rate and reduced the activation energy in the case of Co(1,4-NDC), used here as a model catalyst. The HGR reached 1785.7 mL H₂ g⁻¹ min⁻¹ at room temperature and 6250 mL H₂ g⁻¹ min⁻¹ at 60 °C in the presence of 15 mg NaOH. The activation energy for NaBH₄ hydrolysis over Co(1,4-NDC) decreased from 40.53 to 34.14 kJ/mol in the presence of the base. Moreover, Co(1,4-NDC) demonstrated high recyclability for at least seven cycles, both with and without the base. These synthesized catalysts are considered highly efficient, noble-metal-free catalysts for energy-related applications.

**Supplementary Information:**

The online version contains supplementary material available at 10.1038/s41598-025-27518-4.

## Introduction

Metal–organic frameworks (MOFs) are crystalline porous materials consisting of inorganic nodes (metal ions or clusters) linked together by organic ligands to create extended, periodic architectures. Owing to their well-defined porosity, tunable pore dimensions, and exceptionally high surface areas, MOFs have emerged as a versatile platform for diverse applications, including catalysis^1–4^, adsorption^5,6^, luminescent materials^7,8^, and magnetism^9^.

Hydrogen, a carbon-free energy carrier, is recognized as a renewable, and sustainable energy source^10,11^. It possesses a high gravimetric energy density (142 MJ kg⁻¹), significantly exceeding over three times that of conventional fuels such as diesel and gasoline (46 MJ kg⁻¹), along with high combustion efficiency and environmentally benign by-products^12^. Among the various chemical hydrogen storage materials, boron-based compounds such as ammonia borane (NH₃BH₃) and borohydrides (e.g., LiBH₄ and NaBH₄) are particularly attractive owing to their high hydrogen content^1^. In particular, sodium borohydride (NaBH₄) has emerged as one of the most promising candidates because of its high hydrogen content (10.65 wt%), good stability, low cost, and ease of handling^1,13–15^. Hydrogen can be efficiently generated from NaBH₄ via catalytic hydrolysis under mild conditions, making this route especially attractive for practical applications in on-demand hydrogen generation, according to the following reaction:

1 $${\text{NaB}}{{\text{H}}_{4({\text{aq}})}} + \;\;4{{\text{H}}_{\text{2}}}{{\text{O}}_{({\text{aq}})}}\; \to \;\;{\text{NaB}}{\left( {{\text{OH}}} \right)_{4({\text{aq}})}} + \;\;4{{\text{H}}_{2({\text{g}})}} \uparrow$$

However, the self-hydrolysis of NaBH₄ proceeds very slowly under ambient conditions due to kinetic limitations, necessitating the use of catalysts to achieve practical hydrogen generation rates^1–4,13–17^. Considerable research efforts have therefore focused on developing highly active catalytic systems for this reaction. In our previous work, zirconium-based MOFs linked by 2-amino-terephthalic acid (NH₂-UiO-66) were employed as supports for ruthenium and cobalt nanoclusters to promote hydrogen generation from NaBH₄ hydrolysis^1^. Other reported systems include cobalt nanoparticles anchored on nanoporous graphene oxide^16^ and nickel nanoparticles^17^, both of which have demonstrated effective catalytic activity. Furthermore, the addition of a base, such as NaOH, is commonly used to stabilize NaBH₄ solutions during storage and suppress premature decomposition^18^.

Transition metal-based catalysts such as Co, Ni, Cu, Fe, Zn, Mn and Zr offer a more cost-effective alternative to noble metals; however, their catalytic activity is often inferior, and they typically suffer from poor long-term stability^18–22^. To address these drawbacks, two main approaches have been adopted: (i) enhancing the active sites and surface area while improving catalyst stability, and (ii) designing multi-metallic catalysts that integrate the individual catalytic properties of different metals at the active centers^18–24^. The 1,4-naphthalenedicarboxylic acid (1,4-NDC) linker is particularly well suited for such applications, as it is an aromatic dicarboxylate with distinct structural attributes. Specifically, 1,4-NDC provides excellent bridging capability, high molecular symmetry, and pronounced rigidity due to its naphthalene core, along with versatile coordination modes arising from two carboxylate groups containing four oxygen atoms^25–27^.

For the first time, a series of metal–organic frameworks (Co, Ni, Zr, and Cu) using 1,4-naphthalenedicarboxylic acid (1,4-NDC) as a linker were synthesized for the hydrolysis of NaBH₄ to generate H₂. The synthesized MOFs were analyzed using X-ray diffraction (XRD), transmission electron microscopy (TEM), X-ray photoelectron spectroscopy (XPS), and surface area analysis.

A 50 mg portion of the MOFs [Co(1,4-NDC), Ni(1,4-NDC), UiO-66-1,4-NDC, and Cu(1,4-NDC)] was added to a 50 mM NaBH₄ solution under stirring at a suitable temperature. Within only 5.1 min and 6 min of stirring at room temperature, Co(1,4-NDC) and Ni(1,4-NDC), respectively, achieved the maximum hydrogen volume from NaBH₄ hydrolysis. Increasing the catalyst weight (10–50 mg) and reaction temperature (30–60 °C) gradually enhanced the activity of the synthesized catalysts. The hydrogen generation rate (HGR) values were determined to clarify the effect of each metal cluster in influencing the catalytic performance of NaBH₄ hydrolysis. The effects of adding bases were also investigated, and the rate constant and activation energy were determined in both the presence and absence of the base.

## Experimental

### Chemicals

1,4-naphthalenedicarboxylic acid (1,4-NDC, 98%) linker was purchased from Alfa Aesar. Zirconium chloride (ZrCl4, 98%), N, N-dimethylformamide (DMF, 99%), benzoic acid (PhCOOH, 98%), cobalt nitrate (Co(NO_3_)_2_.6H_2_O, 98%), nickel nitrate (Ni(NO_3_)_2_.6H_2_O, 98%), formic acid (HCOOH, 98%), copper (II) nitrate trihydrate (Cu(NO_3_)_2_·3H_2_O, 98%), sodium hydroxide (NaOH, 97%) and sodium borohydride (NaBH_4_, 98%), methanol (MeOH, 98%) and ethanol (EtOH, 98%) were purchased from Sigma–Aldrich. All chemicals are used as received.

### Synthesis of Co(1,4-NDC) MOF

870 mg of Co(NO_3_)_2_.6H_2_O (3 mmol) and 1.3 g 1,4-naphthalenedicarboxylic acid (NDC) linker (6 mmol) were dissolved in 60 mL of DMF and sealed in a Teflon-lined stainless-steel autoclave followed by heating at 130 °C for 48 h. The mixture was allowed to cool to room temperature, and the violet crystals formed were separated by filtration, then washed with DMF and ethanol. The crystals were subsequently dried at 100 °C for 12 h. The resulting sample was designated as Co(1,4-NDC). The applied method yielded 866 mg of Co(1,4-NDC).

### Synthesis of Ni(1,4-NDC) MOF

Ni(1,4-NDC) was synthesized by dissolving 3.56 g Ni(NO_3_)_2_.6H_2_O and 0.859 g 1,4-naphthalenedicarboxylic acid (NDC) in 80 mL DMF. 14 g Formic acid was added to the mixture as a modulator. The mixture was heated in a 100 mL Teflon-lined stainless-steel autoclave at 130 °C for 8 h. The resulting product was filtered and washed three times with DMF and ethanol, then dried at 100 °C for 12 h. The final sample was designated as Ni(1,4-NDC). The applied method yielded 1.8 g of Ni(1,4-NDC).

### Synthesis of UiO-66-1,4-NDC MOF

UiO-66-1,4-NDC was synthesized as reported before by Butova et al. with modifications^29^. Briefly, 279.6 mg of ZrCl_4_ was suspended in 20 mL DMF, and then 259.4 mg of 1,4-naphthalenedicarboxylic acid (NDC) linker was dissolved in 20 mL DMF and added to the ZrCl_4_ solution. 2.2 g benzoic acid (15 equiv. with respect to the ligand) and 86.7 µL of double distilled water were added to the solution. The mixture was stirred at room temperature for 1 h, then transferred to a 100 mL Teflon-lined stainless-steel autoclave and heated at 120 °C for 48 h under autogenous pressure. The resulting product was filtered using a fine frit and sequentially washed with DMF (2 × 30 mL) and MeOH (2 × 30 mL). Finally, the purified product was dried at 100 °C for 12 h. The obtained sample was designated as UiO-66-1,4-NDC. The applied method yielded 715 mg of UiO-66-1,4-NDC.

### Synthesis of Cu(1,4-NDC) MOF

Cu(1,4-NDC) was denoted as synthesized by solvothermal method as reported before by Arul et al. with modifications^30^. In brief, a mixture of 324 mg of 1,4-naphthalenedicarboxylic acid (NDC) and 362.4 mg of Cu(NO₃)₂·3 H₂O was completely dissolved in 60 mL of DMF and sonicated for 30 min (frequency = 40 kHz; output power = 150 W). The solution was then transferred to a 100 mL Teflon-lined stainless-steel autoclave and heated at 120 °C for 24 h. The resulting product was filtered and washed with DMF and ethanol, followed by drying at 100 °C for 6 h. The final sample was designated as Cu(1,4-NDC). The applied method yielded 380 mg of Cu(1,4-NDC).

### The catalytic hydrolysis of NaBH_4_

The catalytic hydrolysis of NaBH₄ was carried out at 30 °C using 50 mg of the synthesized catalysts (Co(1,4-NDC), Ni(1,4-NDC), UiO-66-1,4-NDC, and Cu(1,4-NDC)). A solution of 94.6 mg of NaBH₄ in 50 mL of double-distilled water (50 mM) was prepared, and 50 mg of each catalyst was introduced into the reaction vessel under continuous stirring at 500 rpm. The volume of hydrogen generated during the reaction was measured using the water displacement method^14,15^.

To investigate the influence of catalyst dosage 10, 30, and 50 mg of the synthesized catalysts were used at 30 °C for 50 mM NaBH_4_ solution.

The synthesized catalysts were used to study the effect of reaction temperature since the reaction was evaluated at different temperatures 30 °C, 40 °C, 50 °C, and 60 °C, over 50 mg of the catalysts and 50 mM NaBH_4_ solution.

To investigate the effect of the base (NaOH), 5, 10, and 15 mg of NaOH were added to a reaction system containing 10 mg of the Co(1,4-NDC) catalyst in a 50 mM NaBH₄ solution at 30 °C. The influence of temperature on the reaction was also examined in the presence of the base.

For the recyclability study, 10 mg of the Co(1,4-NDC) catalyst was tested over seven consecutive cycles for H₂ generation from the 50 mM NaBH₄ solution at 30 °C, both in the absence and presence of the base. After each catalytic run, 50 mM NaBH₄ was introduced into the reaction medium. The spent Co(1,4-NDC) catalyst was subsequently characterized by XRD (Fig. S1) and FTIR (Fig. S2).

## Results and discussion

### Characterization of the synthesized catalysts

**Fig. 1:**
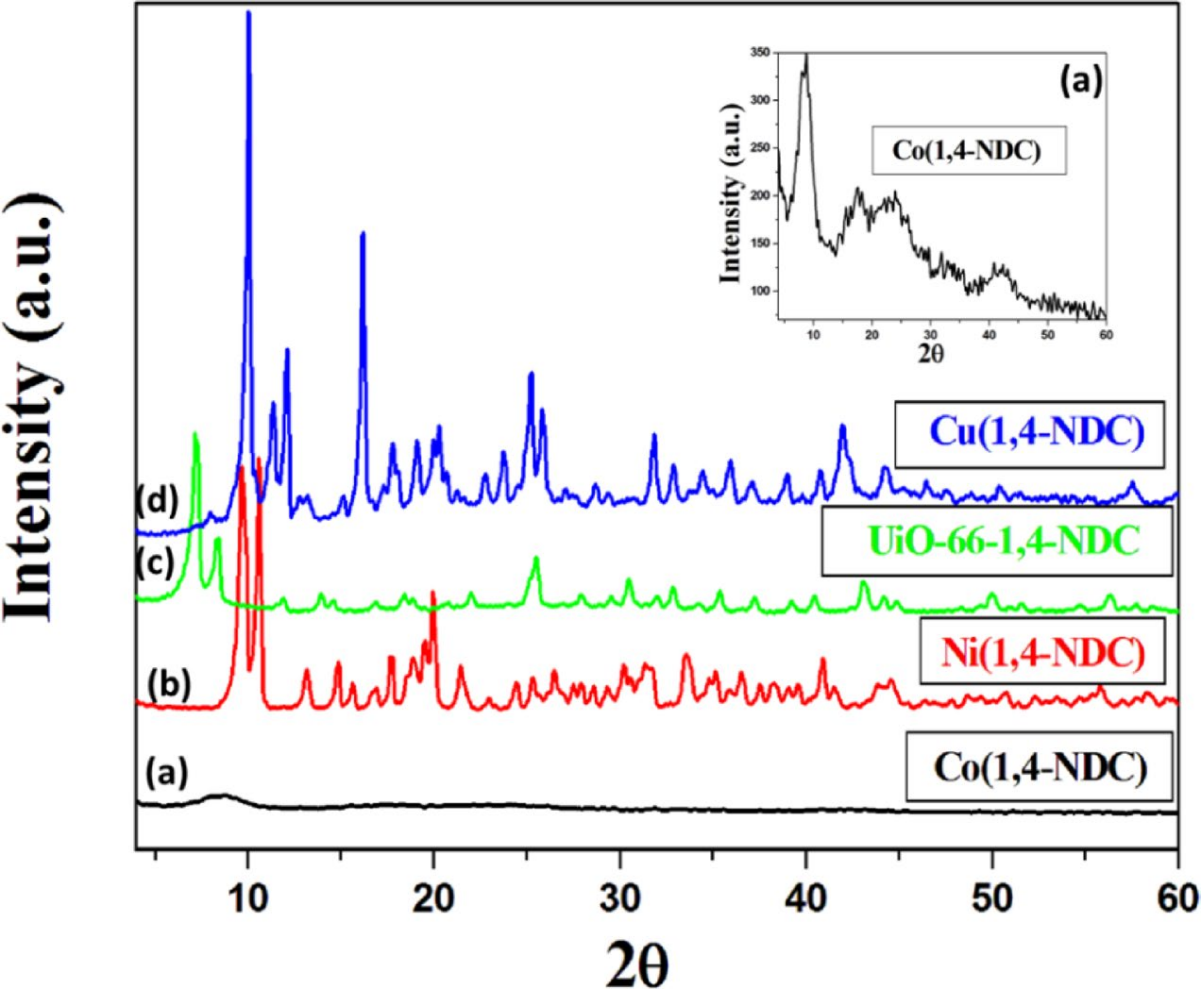
X-ray diffractograms of the synthesized MOFs Co(1,4-NDC), Ni(1,4-NDC), UiO-66-1,4-NDC and Cu(1,4-NDC).

The crystallinity and phase purity of the synthesized MOFs were verified by X-ray diffraction (XRD) analysis. The XRD pattern of Co(1,4-NDC) (Fig. 1a) exhibited a broad characteristic peak at 2θ = 8.8º, consistent with previously reported cobalt-based MOFs^28^. In the case of Ni(1,4-NDC), two sharp reflections appeared at approximately 2θ = 9.7º and 10.6º (Fig. 1b), confirming the successful formation of the MOF structure. The diffraction pattern of UiO-66-1,4-NDC (Fig. 1c) closely resembled that of the parent UiO-66 framework^1^, with a slight shift toward lower 2θ values attributed to an increase in the lattice parameter induced by the additional benzene rings of the 1,4-NDC linker compared with the standard BDC ligand^29^. The simulated patterns of UiO-66-1,4-NDC, Cu(1,4-NDC), Ni(1,4-NDC), and Co(1,4-NDC) MOFs were presented in Fig. S3-S6, respectively. For Cu(1,4-NDC), the XRD pattern (Fig. 1d) displayed intense reflections at 2θ = 10.07º, 16.2º, and 25.22º, corresponding to the (110), (021), and (131) planes, respectively, indicating a high degree of crystallinity. The observed diffraction features of Cu(1,4-NDC) are nearly identical to those of Cu-BDC, in agreement with our previous findings^31^.

**Fig. 2:**
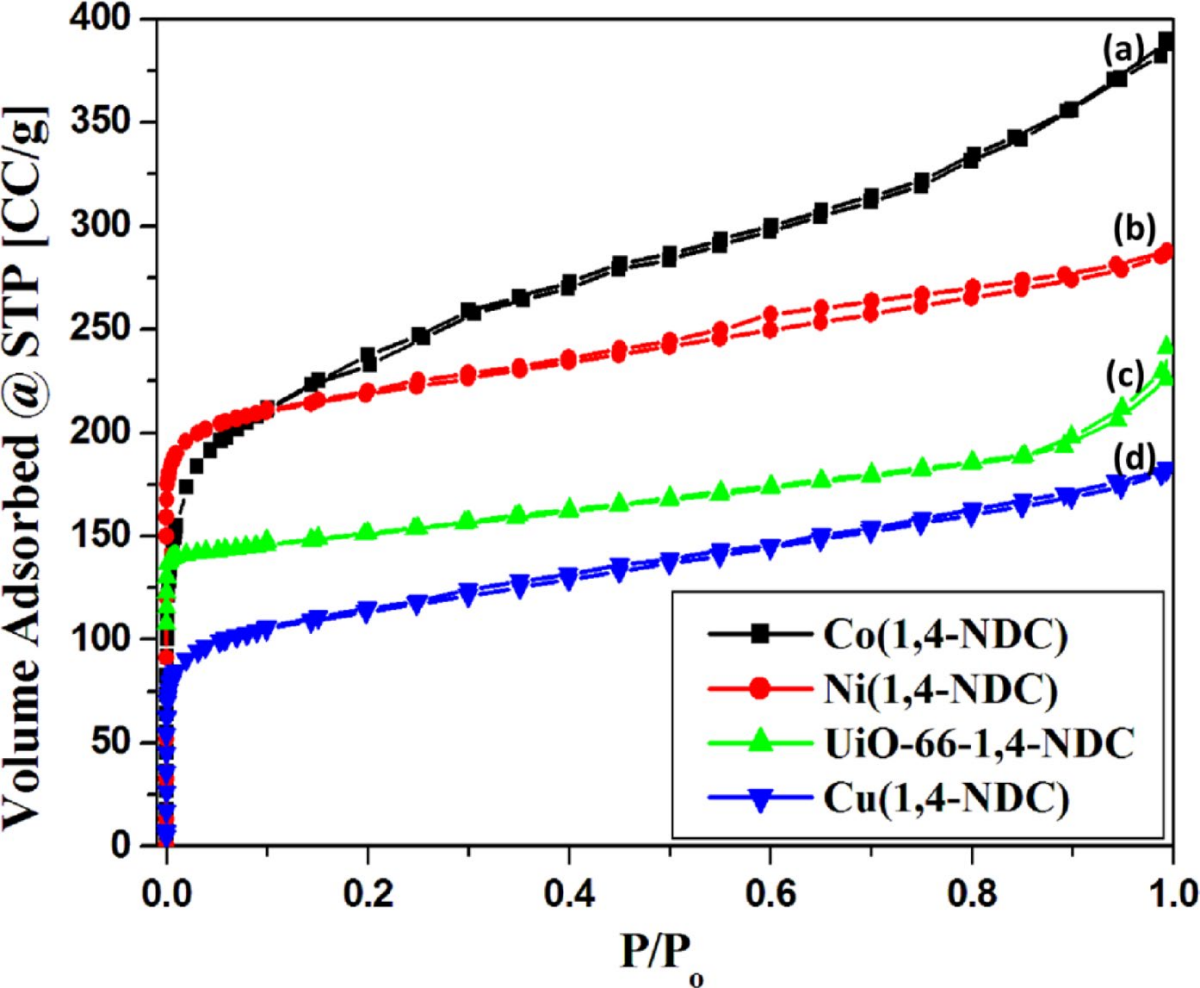
The nitrogen adsorption-desorption isotherms of the synthesized MOFs Co(1,4-NDC), Ni(1,4-NDC), UiO-66-1,4-NDC and Cu(1,4-NDC).

The textural properties of the synthesized MOFs were evaluated by N₂ sorption measurements at 77 K (Fig. 2), with the corresponding parameters summarized in Table 1. The specific surface areas were measured using the Brunauer–Emmett–Teller (BET) method, and the pore size distributions were evaluated using the Barrett–Joyner–Halenda (BJH) method^1–3^. According to IUPAC classification^32^, Cu-, Zr-, and Ni-based MOFs are indeed of type I with some mesoporosity. Nevertheless, the cobalt-based MOF shows gas uptake associated with micropores (up to ~ 150 cm³ g⁻¹) and additional adsorption in the mesoporous region (150–400 cm³ g⁻¹). Therefore, this isotherm cannot be classified solely as type I, but rather as type II as well. This indicates that the material possesses microporosity along with significant mesoporosity (Fig. 2). The Co(1,4-NDC) and Ni(1,4-NDC) samples displayed surface areas of 820 and 770 m²/g, with total pore volumes of 0.45 and 0.4 cm³/g, respectively (Table 1). For UiO-66-1,4-NDC, benzoic acid was employed as a modulator, which enhanced both crystal size and surface area, in agreement with previous reports^29^. Among the investigated MOFs, Cu(1,4-NDC) exhibited the lowest surface area (470 m²/g). The close correspondence between S_BET_ and S_t_ values for all samples confirms the proper choice of standard t-curves for pore analysis confirms the absence of ultra-micropores (Table 1)^33–39^.

**Table 1:** Surface area data for the synthesized catalysts.

Catalysts	S_BET_ (m^2^ g^− 1^)	S_t_ (m^2^ g^− 1^)	Pore volume (cm^3^/g)
Co(1,4-NDC)	820	820	0.45
Ni(1,4-NDC)	770	770	0.4
UiO-66-1,4-NDC	600	600	0.31
Cu(1,4-NDC)	470	470	0.27

**Fig. 3:**
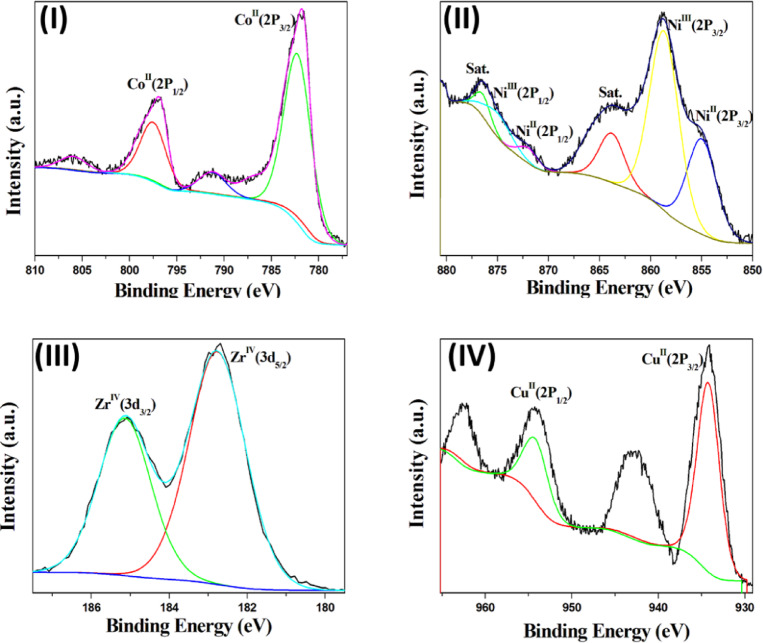
HR-XPS spectra of the (**I**) Co 2P in Co(1,4-NDC), (**II**) Ni 2P in Ni(1,4-NDC), (**III**) Zr 3 d in UiO-66-1,4-NDC and (**IV**) Cu 2P in Cu(1,4-NDC).

X-ray photoelectron spectroscopy (XPS) was used to analyze the chemical composition, oxidation states, and electronic environments of the synthesized MOFs (Fig. 3). The high-resolution Co 2p spectrum of Co(1,4-NDC) (Fig. 3-I) exhibits two main peaks at 782.4 and 797.6 eV, corresponding to Co 2p_3/2_ and Co 2p_1/2_, respectively, with a spin–orbit splitting of 15.2 eV, confirming the predominance of Co²⁺ species^1,40^. Satellite peaks were also observed at ~ 791.3 and 806 eV. For Ni(1,4-NDC) (Fig. 3-II), the Ni 2p spectrum reveals a mixture of Ni²⁺ and Ni³⁺ states. The Ni²⁺ species are identified by peaks at 855.0 and 872.0 eV, while Ni³⁺ is evidenced by additional peaks at 858.0 and 875.3 eV, corresponding to Ni 2p₃_/_₂ and Ni 2p₁_/_₂, respectively. The relative Ni²⁺:Ni³⁺ ratio is estimated at ~ 1:2, with satellite features appearing near 863.8 and 877 eV^41^. The XPS spectrum of UiO-66-1,4-NDC (Fig. 3-III) displays Zr 3d₅_/_₂ and 3d₃_/_₂ peaks at 182.8 and 185.1 eV, consistent with previously reported UiO-66 spectra^31^, confirming Zr in its + 4 oxidation state. For Cu(1,4-NDC) (Fig. 3-IV), the deconvoluted Cu 2p spectrum shows characteristic Cu 2p₃_/_₂ and Cu 2p₁_/_₂ peaks at 934.3 and 954.4 eV, along with shake-up satellites at 942.9 and 962.4 eV, indicative of Cu²⁺ species^30^. Collectively, the XPS results validate the oxidation states and chemical environments of the metal centers in the synthesized MOFs.

**Fig. 4:**
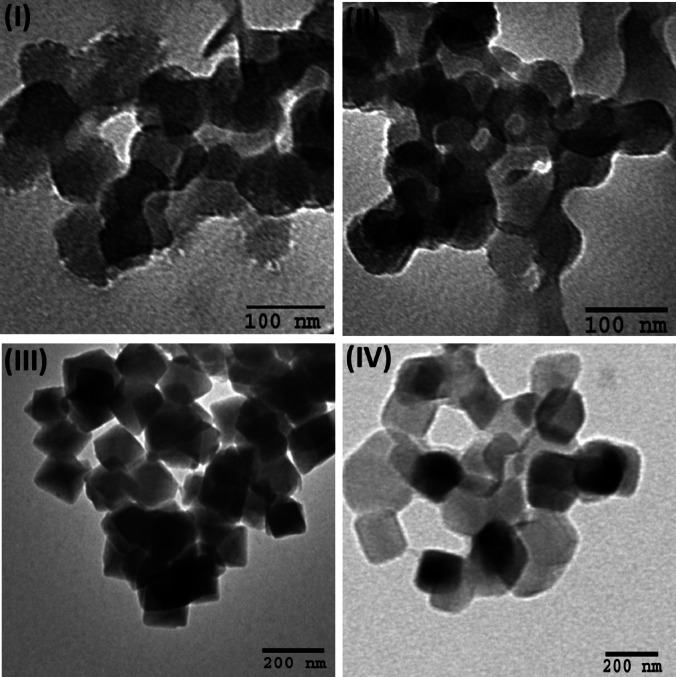
HR-TEM images of (**I**) Co(1,4-NDC) (**II**) Ni(1,4-NDC) (**III**) UiO-66-1,4-NDC and (**IV**) Cu(1,4-NDC) catalysts.

The morphology and particle size of the synthesized MOFs were characterized using transmission electron microscopy (TEM) (Fig. 4)^39,42–44^. Both Co(1,4-NDC) and Ni(1,4-NDC) exhibited relatively small particle sizes, ranging from 100 to 125 nm (Fig. 4-I and 4-II). The UiO-66-1,4-NDC crystals (Fig. 4-III) displayed an average particle size of ~ 150 nm with a well-defined octahedral morphology, closely resembling that of the parent UiO-66 framework^31^. No secondary crystalline phases were observed, confirming the high phase purity of the synthesized material. In contrast, Cu(1,4-NDC) (Fig. 4-IV) exhibited larger crystals with particle sizes distributed between 150 and 250 nm, along with distinct anisotropic structural features^30^.

### Catalytic activity and kinetic measurements

Naphthalenedicarboxylate (NDC)-based MOFs exhibit intrinsic hydrophobicity, well-defined pore geometries, and remarkable thermal stability. These structural attributes not only enable them to serve as storage media for inert gases^45^ but also render certain members suitable for high-energy storage applications^46^. For catalytic processes, the pore dimensions and chemical environment must be optimized to facilitate reactant diffusion and adsorption, while also exhibiting selective affinity toward the products. Several NDC-MOFs fulfill these criteria and have been reported to act as efficient catalysts for specific reactions^47^. Furthermore, extending the π-conjugation within the ligand framework by incorporating additional aromatic rings has been shown to enhance charge transfer through resonance effects, thereby improving both the optical response and catalytic performance of MOFs^48^.

**Fig. 5:**
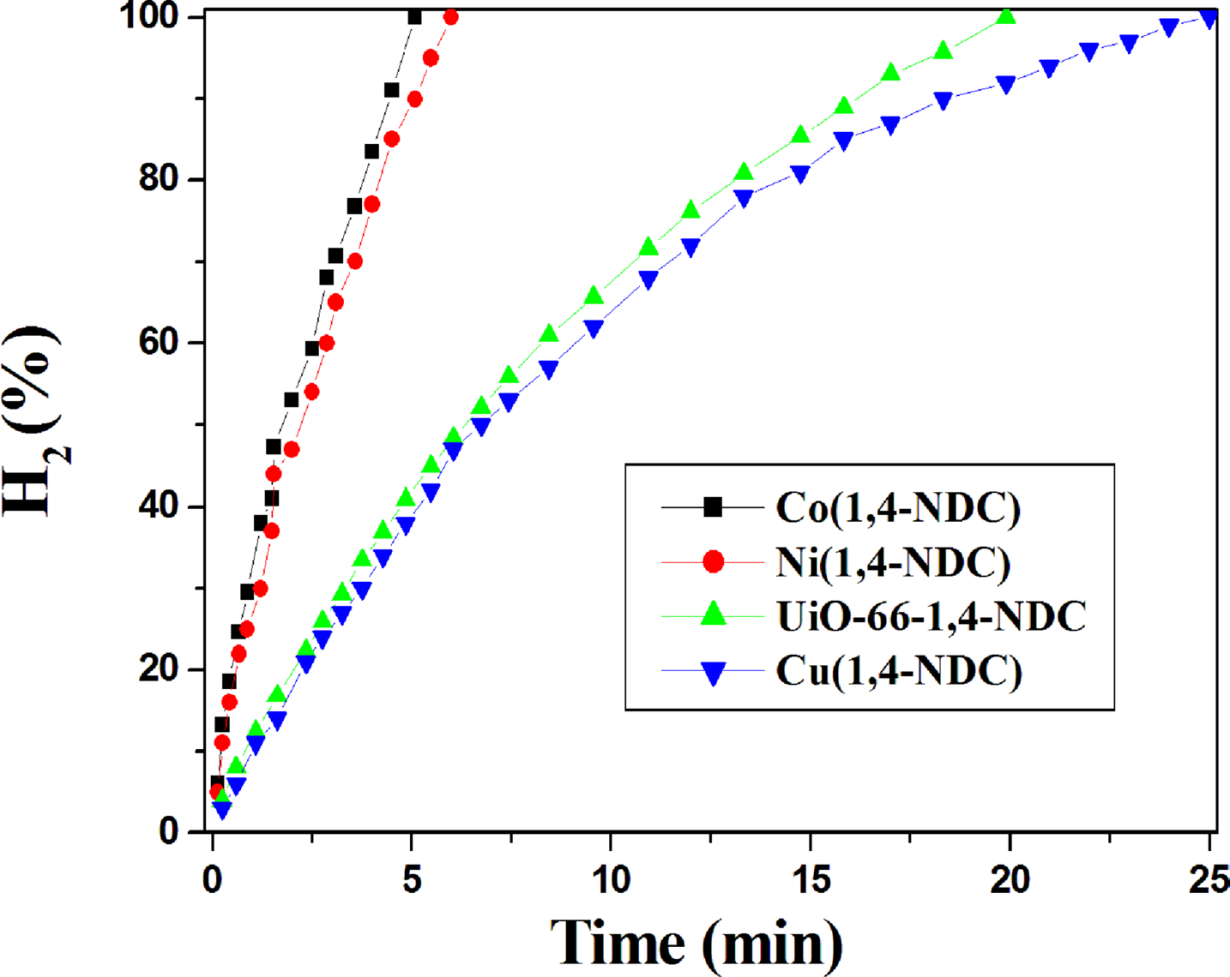
Hydrogen generation from the hydrolysis of NaBH_4_ over the synthesized catalysts Co(1,4-NDC), Ni(1,4-NDC), UiO-66-1,4-NDC and Cu(1,4-NDC). Reaction conditions: 94.6 mg of NaBH_4_ in 50 mL H_2_O (50 mM); Catalyst, 50 mg; at 30 °C.

Figure 5 presents the V_H₂_–t profiles derived from gasometric measurements of NaBH₄ (50 mL, 0.05 M) dehydrogenation at 30 °C in the presence of the synthesized catalysts (50 mg). The plots initially exhibit linear behavior, followed by a downward deviation. The early-stage linearity corresponds to a catalyst surface–dependent hydrogen generation rate (HGR), consistent with zero-order kinetics. The subsequent deviation reflects a reduction in HGR governed by the diminishing reactant concentration, indicative of first-order kinetics. Among the catalysts, Co(1,4-NDC) demonstrates the highest activity, reaching the maximum hydrogen yield within only 5.1 min of stirring at room temperature (Fig. 5). Ni(1,4-NDC), UiO-66-1,4-NDC, and Cu(1,4-NDC) achieve full hydrogen release (75 mL H₂) after 6.0, 19.9, and 25.0 min, respectively. Accordingly, the catalytic activity follows the order: Co(1,4-NDC) > Ni(1,4-NDC) > UiO-66-1,4-NDC > Cu(1,4-NDC) (Fig. 5), which correlates well with the specific surface areas of these MOFs (Table 1). Nevertheless, the self-hydrolysis of the NaBH₄ solution produced 75 mL of H₂ after 58 min of stirring at room temperature.

These findings are consistent with previous reports, such as that by de Oliveira et al.^18^, who demonstrated that a cobalt-based MOF (Co(BDC)) produced 60 mL H₂ within 15 min of stirring at 299 K. By comparison, the present Co(1,4-NDC) catalyst achieved a higher yield (75 mL H₂) in only 5.1 min at room temperature. The superior activity and stability of NDC-based MOFs underscore their promise as efficient catalysts for hydrogen generation, particularly for portable hydrogen fuel systems.

Compared with the commonly used terephthalic acid (BDC), the 1,4-NDC linker affords larger pore sizes and greater π-conjugation. The extended π-conjugated naphthalene core provides enhanced electronic delocalization, which can stabilize the framework and, in some cases, facilitate electron transfer between the linker and metal sites. In addition, 1,4-NDC improves water stability; its larger aromatic surface increases hydrophobic character, thereby enhancing the framework’s resistance to aqueous degradation and promoting more efficient reactant diffusion and catalytic performance in NaBH₄ hydrolysis.

**Fig. 6:**
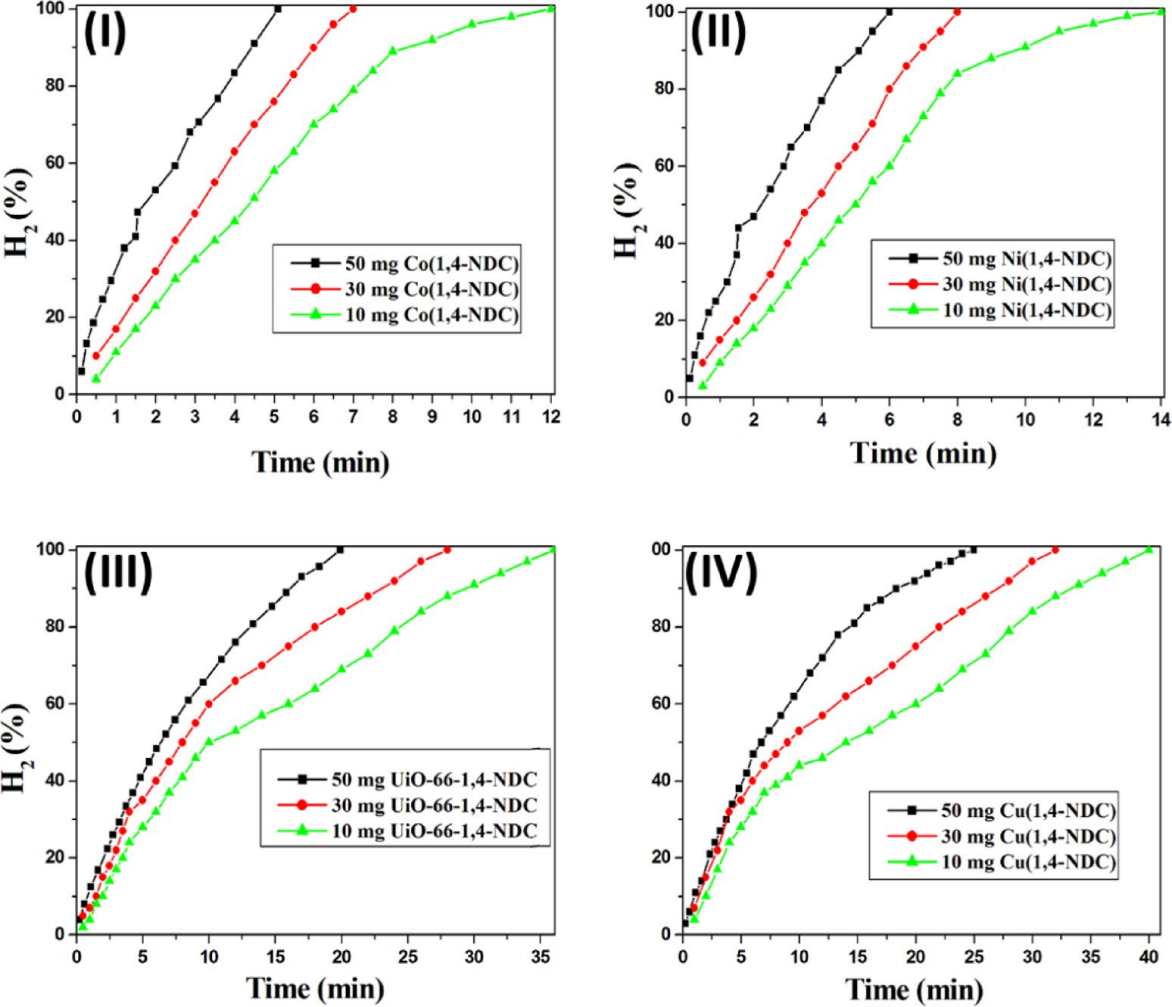
Effect of catalyst weight on hydrogen generation from the hydrolysis of NaBH₄ over (**I**) Co(1,4-NDC), (**II**) Ni(1,4-NDC), (**III**) UiO-66-1,4-NDC and (**IV**) Cu(1,4-NDC). Reaction conditions: 94.6 mg of NaBH_4_ in 50 mL H_2_O (50 mM); Catalyst, 10, 30 and 50 mg; at 30 °C.

The effect of catalyst dosage on hydrogen production was first examined (Fig. 6). Varying amounts of the synthesized catalysts (10, 30, and 50 mg) were introduced into NaBH₄ solution (50 mM) at 30 °C. As shown in Fig. 6, the reaction time decreased with increasing catalyst dosage. This trend indicates that the enhanced hydrogen generation results from increased catalytic surface availability rather than any direct reaction between NaBH₄ and the catalyst itself. For instance, the time required to generate 75 mL of hydrogen decreased from 12.0 to 6.5 and 5.1 min when using 10, 30, and 50 mg of Co(1,4-NDC), respectively (Fig. 6-I). Similar behavior was observed for the other catalysts (Fig. 6).

**Fig. 7:**
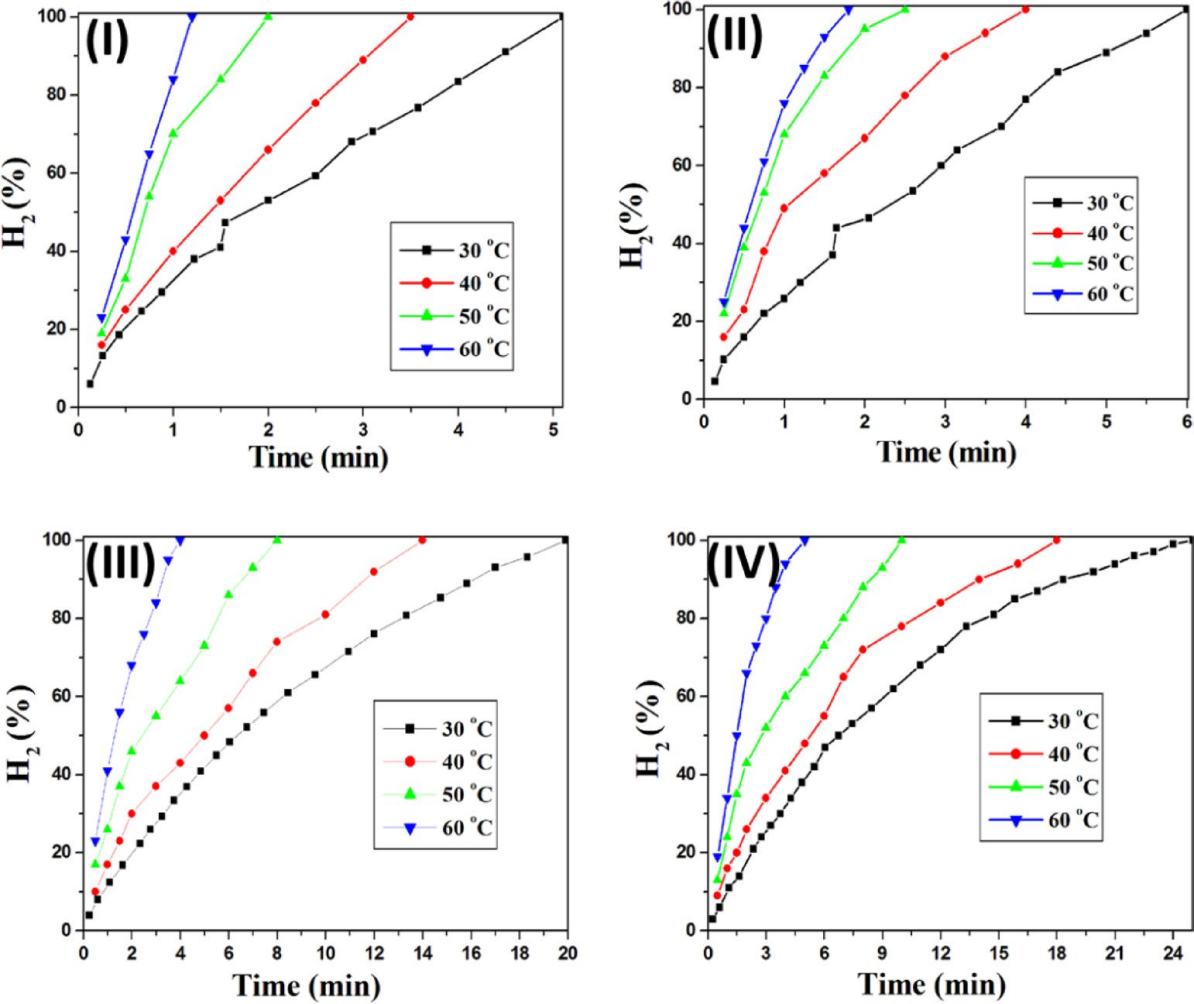
Effect of reaction temperature for hydrogen generation from the hydrolysis of NaBH_4_ over (**I**) Co(1,4-NDC), (**II**) Ni(1,4-NDC), (**III**) UiO-66-1,4-NDC and (**IV**) Cu(1,4-NDC). Reaction conditions: 94.6 mg of NaBH_4_ in 50 mL H_2_O (50 mM); Catalyst, 50 mg; at 30–60 °C.

The effect of reaction temperature was also investigated using 50 mg of each catalyst (Fig. 7). Experiments conducted at 30, 40, 50, and 60 °C revealed that higher temperatures markedly enhanced the catalytic performance of all samples. The most pronounced effect was observed for Co(1,4-NDC), where the time to produce 75 mL of hydrogen decreased from 5.1 min at 30 °C to just 1.2 min at 60 °C. This corresponds to an increase in hydrogen generation rate (HGR) from 294.1 to 1250 mL H_2_ g⁻¹ min⁻¹, representing a 4.25-fold enhancement (Fig. 7-I). Comparable temperature-dependent decreases in reaction time were observed for Ni(1,4-NDC), UiO-66-1,4-NDC, and Cu(1,4-NDC) (Fig. 7-II–IV). Notably, Co(1,4-NDC) and Ni(1,4-NDC) exhibited the highest catalytic efficiencies, producing 200 mL of hydrogen within 2.25 and 3.0 min of stirring at 60 °C, with corresponding HGR values of 1777 and 1333 mL H_2_ g⁻¹ min⁻¹, respectively.

The activation energy (Ea) of the NaBH₄ solution hydrolysis reaction can be determined using the Arrhenius equation^1^ **(**2):

2 $${\text{LnK }} = {\text{ LnA }}-{\text{ }}{{\text{E}}_{\text{a}}}/{\text{RT}}$$

**Fig. 8:**
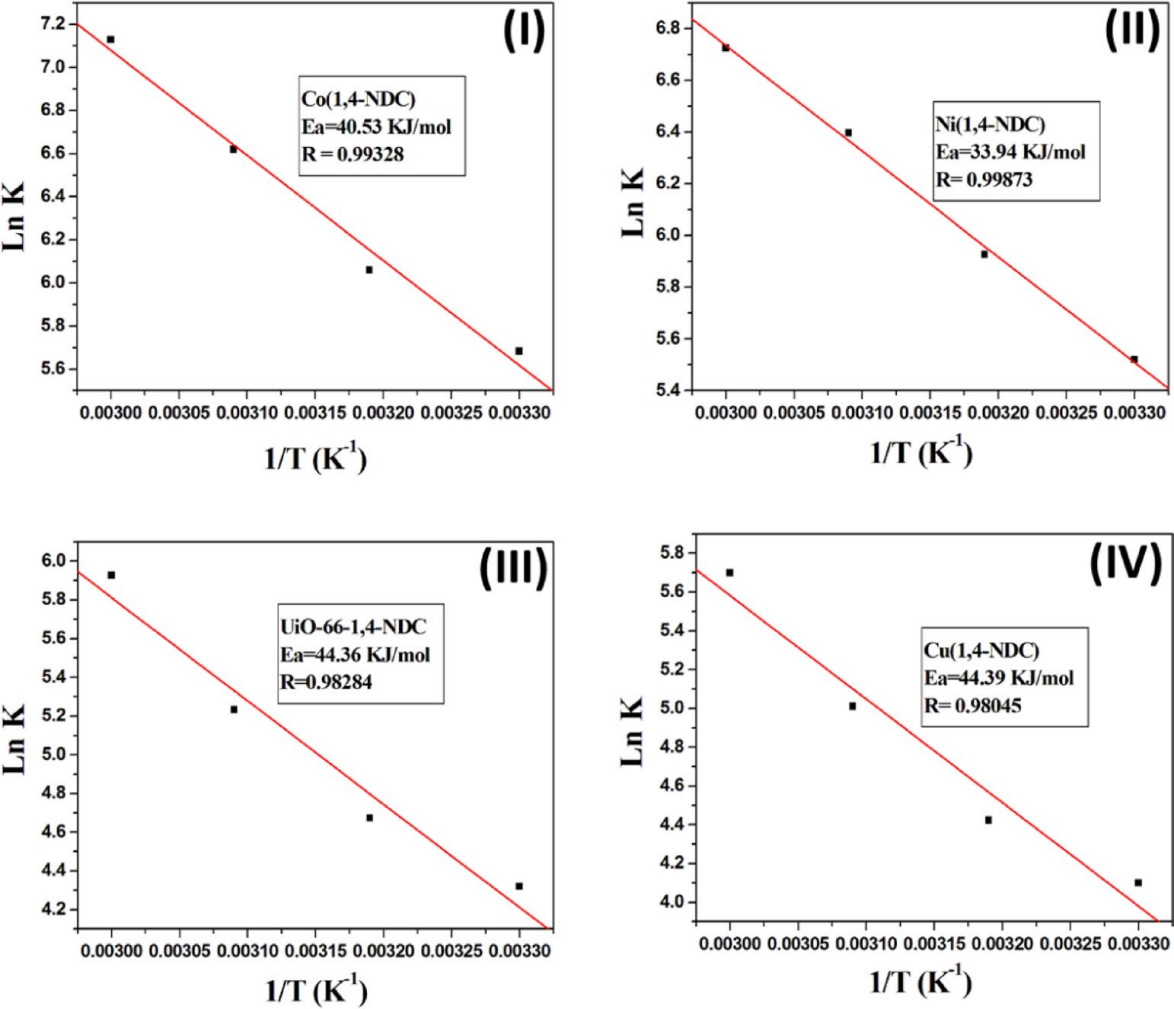
The corresponding Arrhenius plot of ln k versus 1/T illustrating the effect of NaBH₄ solution temperature on hydrogen generation kinetics over the synthesized catalysts (**I**) Co(1,4-NDC), (**II**) Ni(1,4-NDC), (**III**) UiO-66-1,4-NDC and (**IV**) Cu(1,4-NDC).

Where, Ea represents the activation energy (kJ mol⁻¹), R is the gas constant (8.314 J mol⁻¹ K⁻¹), A denotes the pre-exponential factor, T is the solution temperature (K), and k is the rate constant (mL g⁻¹ min⁻¹) measured at different temperatures, which is equal to the hydrogen generation rate (HGR, mL H₂ g⁻¹ min⁻¹). Figure 8 presents the Arrhenius plots (ln k versus 1/T) for Co(1,4-NDC), Ni(1,4-NDC), UiO-66-1,4-NDC, and Cu(1,4-NDC). The corresponding activation energies (Eₐ) were determined to be 40.53, 33.94, 44.35, and 44.39 kJ mol⁻¹, respectively. Notably, the Eₐ values obtained for Co(1,4-NDC) and Ni(1,4-NDC) are lower than those reported for other cobalt- and nickel-based catalysts (Table 2), underscoring their superior catalytic efficiency.

**Table 2:** Comparison of catalysts used in NaBH₄ hydrolysis for hydrogen production.

No.	Catalyst	Activation energy (kJ mol^− 1^)	Ref.
**1**	Co-BDC	43.6	^18^
**2**	Co-B	57.8	^50^
**3**	Co@C	45	^51^
**4**	Co-B(S)	62.4	^52^
**5**	Co-B@TiO_2_	57.0	^53^
**6**	Co@C −700	56.9	^54^
**7**	Co/PGO	55.22	^55^
**8**	Ni_3_B-FeB nanoparticles	40.8	^56^
**9**	NiCoMoMn/Cu	45.3	^57^
**10**	Co-Ni-B	42.8	^58^
**11**	Co(1,4-NDC)	40.53	This work
**12**	Ni(1,4-NDC)	33.94	This work

The catalytic hydrolysis of NaBH₄ over the prepared MOFs most likely proceeds through activation of the borohydride at coordinatively unsaturated metal sites (CUS) located on the metal-cluster nodes. In particular, the metal-cluster nodes (Co, Ni, Zr, or Cu) can act as Lewis-acid sites that coordinate and polarize BH₄⁻, thereby lowering the barrier for B–H bond cleavage and facilitating proton transfer from water to yield H₂. The location of the reaction (within the internal pores or on the external surface) is expected to depend on pore dimensions relative to the reacting species and on the accessibility of active sites. Since NaBH₄ and water are small molecules, they can access internal pore sites in MOFs with micropores or mesopores. Therefore, high surface area alone does not guarantee high activity; pore-size distribution and the presence of mesopores that promote facile mass transport are more critical. For Co(1,4-NDC) and Ni(1,4-NDC), the combination of (i) small particle sizes of the cobalt and nickel clusters in the MOFs, (ii) large surface areas, and (iii) larger pore sizes that enhance reactant diffusion are responsible for their high catalytic activity.

**Fig. 9:**
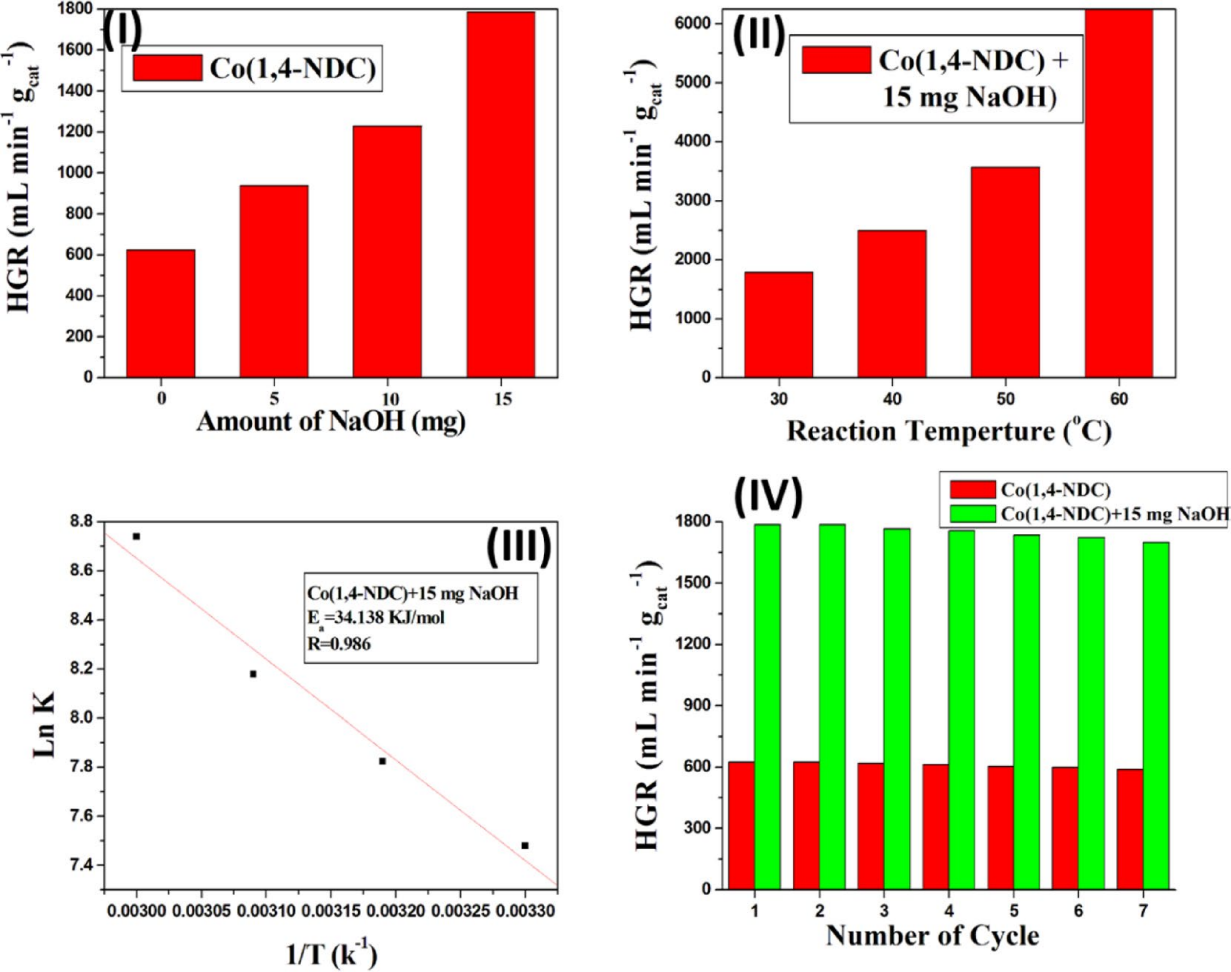
(**I**) Hydrogen generation rate (HGR) from NaBH₄ hydrolysis over the Co(1,4-NDC) catalyst in the presence different amount of NaOH (5, 10 and 15 mg). Reaction conditions: 94.6 mg of NaBH_4_ in 50 mL H_2_O (50 mM); Catalyst, 10 mg; at 30 °C. (**II**) Hydrogen generation rate (HGR) from the hydrolysis of NaBH_4_ over the Co(1,4-NDC) catalyst in the presence of 15 mg NaOH at different reaction temperature. Reaction conditions: 94.6 mg of NaBH_4_ in 50 mL H_2_O (50 mM); Catalyst, 10 mg; at 30–60 °C. (**III**) The corresponding Arrhenius plot of ln k vs. 1/T of effect of NaBH_4_ solution temperature on hydrogen generation kinetics over Co(1,4-NDC) and 15 mg NaOH. (**IV**) Recyclability effect of Co(1,4-NDC) in absence and presence of 15 mg NaOH. Reaction conditions: 94.6 mg of NaBH_4_ in 50 mL H_2_O (50 mM); Catalyst, 10 mg; at 30 °C.

It is well established that storing NaBH₄ in aqueous solutions under alkaline conditions suppresses uncontrolled self-hydrolysis^49^. Accordingly, catalysts designed for NaBH₄ hydrolysis must remain effective under such conditions. Furthermore, the hydrolysis of NaBH₄ under basic conditions follows a zero-order kinetic model^18^. To examine this, sodium hydroxide was introduced into the reaction system^18^, and Co(1,4-NDC), the most active catalyst, was selected for further evaluation. As shown in Fig. 9-I, the catalytic performance of Co(1,4-NDC) improved progressively with the addition of 5, 10, and 15 mg NaOH. At room temperature, the hydrogen generation rate (HGR) increased from 625 mL H_2_ g⁻¹ min⁻¹ in the absence of base to 1785.7 mL H_2_ g⁻¹ min⁻¹ in the presence of 15 mg NaOH. The corresponding rate constant (k) for Co(1,4-NDC) was nearly three times greater than the value obtained without NaOH (Fig. 9-I).

Based on the optimization study, 15 mg of NaOH was identified as the optimal base concentration and subsequently employed to investigate the temperature effect. As shown in Fig. 9-II, increasing the reaction temperature from 30 to 60 °C significantly enhanced hydrogen generation over Co(1,4-NDC) in the presence of the base. The HGR increased steadily with temperature, reaching a maximum value of 6250 mL H_2_ g⁻¹ min⁻¹ at 60 °C. Furthermore, the addition of NaOH decreased the activation energy of Co(1,4-NDC) from 40.53 kJ mol⁻¹ (without base) to 34.14 kJ mol⁻¹ (with 15 mg NaOH), confirming that the base promotes a more favorable reaction pathway (Fig. 9-III).

In the hydrolysis of NaBH₄, the addition of a base plays a critical role in regulating the reaction. The alkaline medium suppresses the spontaneous self-hydrolysis of NaBH₄ by stabilizing the BH₄⁻ anion and minimizing proton availability, thereby preventing uncontrolled hydrogen release in the absence of a catalyst. Moreover, the basic environment maintains the solubility of the hydrolysis by-products as [B(OH)₄]⁻, reducing catalyst deactivation caused by insoluble borate precipitates. The base also enhances catalyst stability by inhibiting metal leaching and surface oxidation under aqueous conditions. Consequently, the presence of a base allows for controlled hydrogen generation, with the reaction rate predominantly governed by the catalyst rather than non-catalytic decomposition.

To further assess the practical applicability, the catalytic stability of Co(1,4-NDC) was evaluated through multi-cycle experiments at room temperature. Given its superior hydrogen generation performance, Co(1,4-NDC) was chosen as the representative catalyst. Reusability tests were conducted both in the absence and presence of NaOH, without catalyst regeneration between cycles^1,18^. As shown in Fig. 9-IV, the HGR values remained nearly constant across seven consecutive cycles, irrespective of the reaction medium. These results demonstrate the excellent stability and recyclability of Co(1,4-NDC), highlighting its potential as a robust catalyst for sustainable hydrogen generation. XRD and FT-IR analyses of Co(1,4-NDC) were performed before and after the reaction. As shown in Fig. S1 and Fig. S2, the chemical structure of Co(1,4-NDC) remained unchanged after the reaction.

### Comparison with other catalysts used for hydrogen generation

A comparative analysis of cobalt- and nickel-based catalysts previously reported for NaBH₄ hydrolysis is summarized in Table 2. To assess the catalytic efficiency of the prepared materials, the activation energy values of Co(1,4-NDC) and Ni(1,4-NDC) were benchmarked against those of other cobalt- and nickel-based catalysts^50–58^. As shown in Table 2, the activation energies associated with Co(1,4-NDC) and Ni(1,4-NDC) are significantly lower than those reported for most analogous materials in the literature, highlighting the superior catalytic activity of the synthesized MOFs toward NaBH₄ hydrolysis.

## Conclusion

In summary, four MOFs Co(1,4-NDC), Ni(1,4-NDC), UiO-66-1,4-NDC, and Cu(1,4-NDC) were successfully synthesized via a simple one-step solvothermal method and obtained as phase-pure crystalline products, as confirmed by PXRD analysis. These MOFs were employed as catalysts for NaBH₄ hydrolysis at room temperature to generate hydrogen as an efficient energy source. Among them, Co(1,4-NDC) demonstrated the highest catalytic performance, attributed to its smallest particle size, largest surface area, and highest pore volume. The Co(1,4-NDC) achieved hydrogen generation rate (HGR) of 625 mL H₂ g⁻¹ min⁻¹ at room temperature. Systematic studies on catalyst loading and reaction temperature (30–60 °C) revealed that Co(1,4-NDC) and Ni(1,4-NDC) achieved remarkable hydrogen generation rates (HGRs) of 1777 and 1333 mL H₂ g⁻¹ min⁻¹ at 60 °C, respectively. The apparent activation energy for NaBH₄ hydrolysis over Ni(1,4-NDC) was determined to be 33.94 kJ/mol, which is lower than that reported for many MOFs employing other linkers. Furthermore, the introduction of a small amount of NaOH significantly enhanced the catalytic activity of Co(1,4-NDC), increasing the HGR to 1785.7 mL H₂ g⁻¹ min⁻¹ at room temperature and 6250 mL H₂ g⁻¹ min⁻¹ at 60 °C. Correspondingly, the activation energy decreased from 40.53 to 34.14 kJ mol⁻¹ in the presence of base. The Co(1,4-NDC) catalyst also exhibited excellent durability, maintaining stable activity over at least seven consecutive cycles at room temperature without regeneration. Considering its low-cost precursors, noble-metal-free composition, and facile synthesis, Co(1,4-NDC) emerges as a highly promising candidate for practical hydrogen generation and future industrial energy applications.

## Supplementary Information

Below is the link to the electronic supplementary material.

Supplementary Material 1

## Data Availability

All data generated or analyzed during this study are included in this published article (and its supplementary information files).
