# Supplementary material for: Synthesis and characterization of Co, Ni, Zr and Cu MOFs based on 1,4-naphthalenedicarboxylic acid linker for hydrogen generation
Source: Sci Rep. 2025 Nov 25;15:42014. doi: 10.1038/s41598-025-27518-4 (PMC12657995; doi:10.1038/s41598-025-27518-4)
Supplement: Supplementary file 1 — Supplementary Material 1 [file 41598_2025_27518_MOESM1_ESM.docx]

**Supplementary Information**

**Synthesis and characterization of Co, Ni, Zr and Cu MOFs based on 1,4-naphthalenedicarboxylic acid linker for hydrogen generation**

**Mostafa Farrag***

Nanoclusters and Photocatalysis Laboratory, Chemistry Department, Faculty of Science, Assiut University, 71516 Assiut, Egypt

*[mostafafarrag@aun.edu.eg](mailto:mostafafarrag@aun.edu.eg)

**Instrumentation and characterization**

Adsorption–desorption isotherms of liquid nitrogen at −196 ^o^C are obtained using a Quantachrome (Nova 3200 series) multi-gas adsorption apparatus. Prior to analysis, the samples were outgassed at 150 ^o^C for 3 hrs. Specific surface areas are calculated from these isotherms by applying the BET equation. S_t_ Values are calculated using the V_a–t_ plots of de Bore. Powder X-ray diffraction (XRD) is performed on a Philips X-ray powder diffractometer, model pw 2013/00. Ni-filtered Cu Kα with a wavelength of λ = 1.541838 Å was used as a constant source of radiation. The generator was operated at 35 kV and 20 mA, and diffractometer at 50 diverting and receiving slits and a scan rate of 20 mm/min. Fine powder samples were loaded on a quartz plate holder by spreading the powders as a smooth thin layer on the plate. For all diffractograms, the following settings were used: scan range 2–60^o^ (2θ), scan step 0.06^◦^. The surface electronic states are investigated by using XPS K-ALPHA (Themo Fisher Scientific, USA) with monochromatic X-ray Al K-alpha radiation -10 to 1350 e.v spot size 400 micro m at pressure 10^-9^ mbar with full-spectrum pass energy 200 e.v and narrow-spectrum 50 e.v. The XPS data are calibrated internally by fixing the binding energy (BE) of the C1s peak at 284.6 eV. For TEM measurements, solutions with a concentration of 1−2 mg/mL are prepared by dissolving the samples in 2^nd^ distilled water. A droplet of these samples solutions is casted onto carbon-coated copper grids. The solvent is then allowed to evaporate slowly. HR-TEM images are obtained with a JEOL JEM 2010 with LaB6-Cathode electron microscope operating at an acceleration voltage of 200 kV. The images are then analyzed by using Image J software (version 1.44). iCAP 6000 Series ICP Emission Spectrometer – Thermo was used to determine the loaded Ru and Co nanoclusters.

**

**

**Fig. S1**. X-ray diffractograms of the Co(1,4-NDC) before and after the hydrolysis of NaBH_4_ reaction.





**Fig. S2**. FTIR analysis of the Co(1,4-NDC) before and after the hydrolysis of NaBH_4_ reaction.


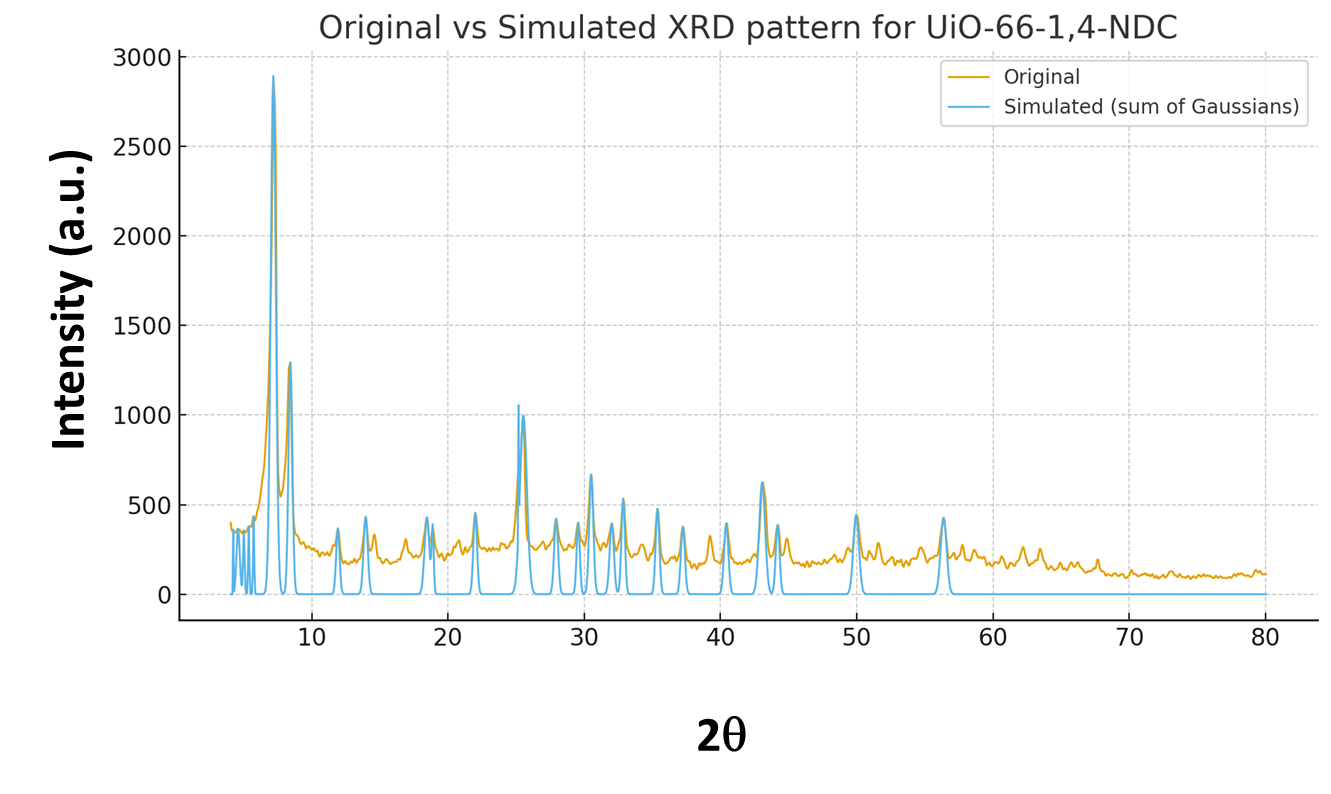


**Fig. S3**: Simulated XRD pattern of UiO-66-1,4-NDC.





**Fig. S4**: Simulated XRD pattern of Cu-1,4-NDC.





**Fig. S5**: Simulated XRD pattern of Ni-1,4-NDC.





**Fig. S6**: Simulated XRD pattern of Co-1,4-NDC.
